# Benchmarking the Self-Assembly of Surfactin Biosurfactant at the Liquid–Air Interface to those of Synthetic Surfactants

**DOI:** 10.1007/s11743-016-1796-9

**Published:** 2016-02-27

**Authors:** Sagheer A. Onaizi, M. S. Nasser, Nasir M. A. Al-Lagtah

**Affiliations:** School of Chemical Engineering and Advanced Materials, Newcastle University, 537 Clementi Road, #06-01, Singapore, 599493 Singapore; School of Chemical Engineering and Advanced Materials, Newcastle University, Newcastle upon Tyne, NE1 7RU UK; Gas Processing Center, College of Engineering, Qatar University, P. O. Box 2713, Doha, Qatar

**Keywords:** Adsorption, Surfactin, Biosurfactant, Synthetic surfactant, Anionic, Nonionic, Self-assembly, Liquid–air interface

## Abstract

The adsorption of surfactin, a lipopeptide biosurfactant, at the liquid–air interface has been investigated in this work. The maximum adsorption density and the nature and the extent of lateral interaction between the adsorbed surfactin molecules at the interface were estimated from surface tension data using the Frumkin model. The quantitative information obtained using the Frumkin model was also compared to those obtained using the Gibbs equation and the Langmuir–Szyszkowski model. Error analysis showed a better agreement between the experimental and the calculated values using the Frumkin model relative to the other two models. The adsorption of surfactin at the liquid–air interface was also compared to those of synthetic anionic, sodium dodecylbenzenesulphonate (SDBS), and nonionic, octaethylene glycol monotetradecyl ether (C_14_E_8_), surfactants. It has been estimated that the area occupied by a surfactin molecule at the interface is about 3- and 2.5-fold higher than those occupied by SDBS and C_14_E_8_ molecules, respectively. The interaction between the adsorbed molecules of the anionic biosurfactant (surfactin) was estimated to be attractive, unlike the mild repulsive interaction between the adsorbed SDBS molecules.

## Introduction

Sustainable and more environmentally-friendly technologies have been (and are still) actively sought to replace several conventional ones. For example, more clean and sustainable surface active agents (i.e., biosurfactants) have emerged as alternatives to synthetic surfactants, which are derived from polluting and unsustainable fossil fuels, in several applications [1]. Some of these bio-based surfactants have shown superior interfacial activities (e.g., reduction of interfacial tension of fluid–fluid interfaces [2, 3]) relative to synthetic ones. A recent study has also demonstrated that a detergent formulation containing biosurfactant is more effective in cleaning protein stains from solid surfaces relative to other formulations containing synthetic surfactants [4]. Additionally, a self-assembled thin film at the liquid–air interface from a binary mixture of biosurfactant–synthetic surfactant had a biosurfactant fraction that is more than 5-fold higher than its fraction in the binary mixture [5]. Thus, biosurfactants are promising surface active agents with a wide scope of industrial applications. The key obstacle for the full utilization of biosurfactants is their current high manufacturing cost, relative to the synthetic ones. However, as it is the usual case with any new technology, this limitation will be overcome in future with further advancement in the biosurfactant production and purification techniques.

In addition to the advancement in biosurfactant manufacturing technology, the full utilization of biosurfactants requires the understanding of their interfacial behavior and also the benchmarking of such behavior to that of synthetic surfactants. Such understanding and benchmarking are still greatly lacking. Thus, the aim of this study is to investigate the adsorption of surfactin, which is an interesting lipopeptide biosurfactant, at liquid–air interface; the adsorption of this biosurfactant will be compared to those of SDBS (anionic) and C_14_E_8_ (nonionic) synthetic surfactants. Unlike the limited number of published studies on biosurfactant adsorption, the adsorption of synthetic surfactants at different interfaces has been widely studied (see for examples [6–12]). Adsorption from synthetic surfactants [7, 13, 14] or protein-surfactant [15, 16] mixtures has been also addressed in some of the previously published studies. Additionally, a few studies have been conducted to investigate the adsorption of synthetic surfactant–biosurfactant mixtures at liquid–air interfaces [5, 17, 18]. Nonetheless, to the best of the authors’ knowledge, quantitative benchmarking of biosurfactant adsorption to those of synthetic ones under the same experimental conditions has not yet been established in the literature.

The adsorption of surfactin (and the other two synthetic surfactants) takes place from solutions containing high concentrations of co- and counter-ions as such a condition is of more industrial relevance [19]. The self-assembly of these surfactants at the liquid–air interface will be followed using surface tension measurements and the obtained data will be analyzed theoretically using appropriate models to extract quantitative information on the adsorption process. Error analysis will be performed in order to assess the agreement between the computed values using the different (Frumkin, Gibbs equation and the Langmuir–Szyszkowski) models and the measured ones. Furthermore, the nature (i.e., attractive or repulsive) and also the extent of interaction between the adsorbed surfactant molecules at the liquid–air interface will be reported.

## Materials and Methods

The biosurfactant, surfactin, was purchased from Wako Pure Chemical Industries Ltd (Japan). Sodium dodecylbenzenesulphonate and octaethylene glycol monotetradecyl ether were purchased from Sigma–Aldrich. Solutions of different surfactant concentrations were prepared by dissolving the surfactant of interest in 20 mM sodium phosphate buffer at pH 8. The buffer was made by dissolving the required amounts of monosodium phosphate (NaH_2_PO_4_) and disodium phosphate (Na_2_HPO_4_), which are both of analytical grade, in demineralized and purified water using a Millipore water purification system. The self-assembly of the three surface active agents at the liquid (buffer)–air interface was followed using a DSA10 tensiometer (Krüss GmbH, Hamburg, Germany). This was achieved by creating ~8 μL pendant air bubble in 8 mL of the surfactant solution of interest and then following the time-dependant reduction in the interfacial tension of the liquid–air interface until an equilibrium surface tension ($\gamma^{e}$) value is reached. The above procedure was repeated for other surfactant concentrations ($C$) in order to obtain sets of $\gamma^{e} - C$ data for the three surfactants. All surface tension measurements (performed in triplicate) were carried out at a fixed temperature of ~25 °C. The reproducibility of $\gamma^{e}$ was quite high in which the variations in $\gamma^{e}$ between the different runs at each surfactant concentration never exceeded 3 %.

The $\gamma^{e} - C$ data in the premicellar region were further analysed to extract quantitative information on the adsorption of the three surfactants at the liquid–air interface. The most important quantitative parameter that can be extracted from the adsorption process is the maximum adsorption density ($\varGamma_{\infty }$). To enable the estimation of $\varGamma_{\infty }$ from the surface tension data, the equilibrium surface tensions at different bulk concentrations of the three surface active agents in the premicellar region were regressed using different models. The simplest way to estimate $\varGamma_{\infty }$ is to plot $\gamma^{e}$*versus* the logarithmic values of $C$ up to the critical micelle concentration (CMC) of the surfactant. The slope can be used to estimate $\varGamma_{\infty }$ according to the Gibbs adsorption isotherm shown in Eq. 1 [20, 21]:

1 $$\left( {\frac{{\partial \gamma^{e} }}{\partial \ln C}} \right)_{T} = - n{\text{RT}}\,\varGamma_{\infty }$$

where *T* is the absolute temperature, *R* is the universal gas constant and *n* is a prefactor, which is 1 for nonionic surfactants such as C_14_E_8_. For ionic surfactants (e.g., surfactin and SDBS), the value of *n* depends on the number of species produced from the dissociation of each surfactant molecule and also on the type and concentration of the counter-ion(s) co-existing with the surfactant molecules in the solution [22–24]. When ionic surfactants co-exist with a relatively higher concentration of the counter-ion(s), as it is the case in this study, the value of *n* approaches unity [10, 19, 25].

The simplicity of Eq. 1 has attracted several researchers to use it for the estimation of $\varGamma_{\infty }$ for different surfactants adsorbing at fluid–fluid interfaces. Another model that can be used to estimate $\varGamma_{\infty }$ from the equilibrium surface tension data is the Langmuir–Szyszkowski model [26–28] (Eq. 2), which is derived from the Gibbs equation of state coupled with the Langmuir adsorption isotherm [29–31]:

2 $$\gamma^{e} = \gamma_{0} - {\text{RT}}\,\varGamma_{\infty } \ln \left( {1 + {\text{KC}}} \right)$$

where $\gamma_{0}$ and $K$ are the surface tension of the solvent in the absence of surfactant and the adsorption equilibrium constant, respectively. Although the Langmuir–Szyszkowski model has been widely used to estimate $\varGamma_{\infty }$, it does not take into account the interaction between the adsorbed molecules at the interface, which is an obvious limitation, particularly for ionic surfactants. Such a limitation is addressed by the Frumkin model [10, 32, 33] which accounts for the lateral interaction between the adsorbed molecules, shown in the coupled Eqs. 3 and 4:

3 $$\gamma^{e} = \gamma_{0} + \frac{{{\text{RT}}\,\varGamma_{\infty } }}{2}\left( {2\ln \left( {1 - \varGamma^{e} /\varGamma_{\infty } } \right) + \beta \left( {\varGamma^{e} /\varGamma_{\infty } } \right)^{2} } \right)$$

4 $$C = \frac{{\varGamma^{e} \exp \left( { - \beta \,\varGamma^{e} /\varGamma_{\infty } } \right)}}{{K\left( {\varGamma_{\infty } - \varGamma^{e} } \right)}}$$

where $\varGamma^{e}$ and *β* are the equilibrium surface coverage at a given surfactant bulk concentration and the lateral interaction parameter between the interfacially adsorbed surfactant molecules, respectively.

## Results and Discussion

**Fig. 1:**
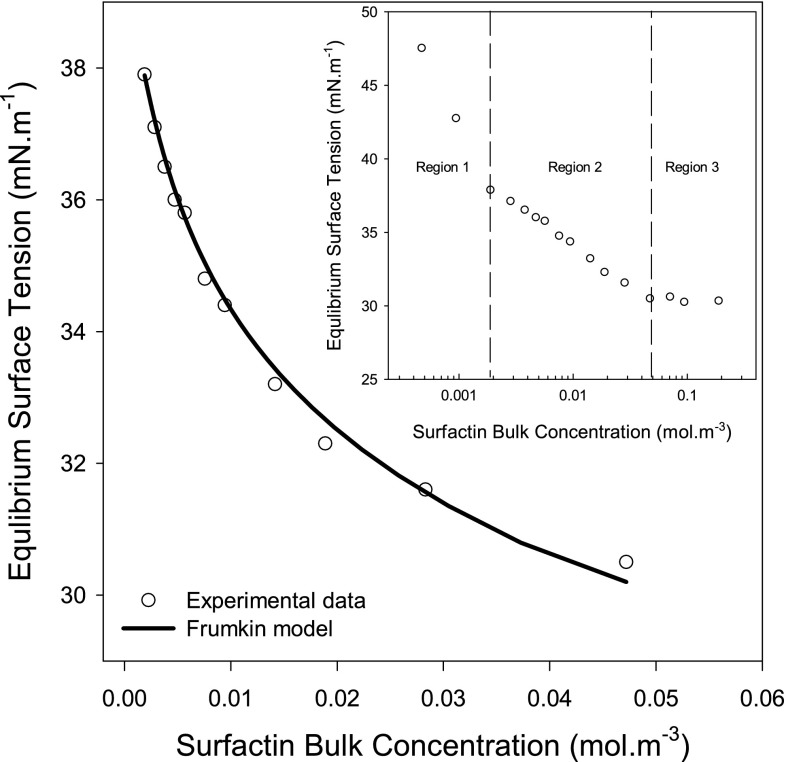
Regression of surfactin $\gamma^{e} - C$ data in region 2 using the modified Frumkin model (the coupled Eqs. 5 and 6). The estimated maximum adsorption density ($\varGamma_{\infty }$) and the area occupied by a surfactin molecule at the liquid–air interface are shown in Table 1. The *inset* is the plot of $\gamma^{e} - \ln C$ data

The Frumkin model was used to fit the premicellar $\gamma^{e} - C$ data of surfactin. However, the regression of the entire premicellar $\gamma^{e} - C$ region using the Frumkin model was quite poor (results not shown). The plot of $\gamma^{e} - \ln C$ (see Fig. 1 inset) has two distinct slopes in the premicellar region. Owing to the high surface activity of surfactin, the adsorption of the biosurfactant at the liquid–air interface from relatively low bulk concentrations (e.g., region 1) leads to the depletion of the biosurfactant molecules from the solution, resulting in a steep slope of the $\gamma^{e} - \ln C$ plot. To have reliable estimates of surfactin adsorption parameters, only $\gamma^{e} - C$ data in region 2 were regressed using the modified (to account for the new boundary conditions) Frumkin model shown in Eqs. 5 and 6.

5 $$\gamma_{2}^{e} = \gamma_{1 - 2}^{e} + \frac{{{\text{RT}}\varGamma_{\infty } }}{2}\left( {2\ln \left( {\frac{{\varGamma_{\infty } - \varGamma^{e} }}{{\varGamma_{\infty } - \varGamma_{1 - 2}^{e} }}} \right) + \beta \left( {\left( {\frac{{\varGamma^{e} }}{{\varGamma_{\infty } }}} \right)^{2} - \left( {\frac{{\varGamma_{1 - 2}^{e} }}{{\varGamma_{\infty } }}} \right)^{2} } \right)} \right)$$

6 $$C_{2} = C_{1 - 2} + \frac{1}{K}\left( {\frac{{\varGamma^{e} \exp \left( { - \beta \varGamma^{e} /\varGamma_{\infty } } \right)}}{{\varGamma_{\infty } - \varGamma^{e} }} - \frac{{\varGamma_{1 - 2}^{e} \exp \left( { - \beta \varGamma_{1 - 2}^{e} /\varGamma_{\infty } } \right)}}{{\varGamma_{\infty } - \varGamma_{1 - 2}^{e} }}} \right)$$

The subscript 2 refers to the data in region 2 while the symbols $\gamma_{1 - 2}^{e}$, $C_{1 - 2}$, $\varGamma_{1 - 2}^{e}$ refer to the equilibrium surface tension, surfactin bulk concentration and equilibrium surface coverage, respectively, at the intersection of region 1 and 2.

The regression of $\gamma_{2}^{e} - C_{2}$ data using the modified Frumkin model is shown in Fig. 1. The estimated maximum adsorption density ($\varGamma_{\infty }$) is 1.16 µmol m^−2^, corresponding to an area per surfactin molecule at the liquid–air interface of 143 Å^2^. This surfactin molecular area is very similar to that (147 ± 5 Å^2^) reported by Li *et al*. [34] for surfactin adsorption at the liquid–air interface using neutron reflectivity (NR). Shen *et al*. [35] also reported a comparable molecular area (147 and 150 Å^2^ at pH 7.5 and 8.5, respectively) for surfactin at the liquid–air interface. Furthermore, molecular dynamic simulation studies [36, 37] reported a molecular area for surfactin monolayer at the liquid–air interface in the range of 126–170 Å^2^. Generally, most of the published studies on surfactin adsorption at the air–water interface report a limiting molecular area ranging from 126 to 220 Å^2^ [36, 38]. The area occupied by a surfactin molecule at the liquid–air interface reported in this work falls within this range.

In addition to the Frumkin model, surfactin $\gamma^{e} - C$ data in region 2 were also regressed using the Gibbs equation (Eq. 1) and a modified (after taking the change in the boundary conditions into account) Langmuir–Szyszkowski model [19]. Although the Gibbs equation and the modified Langmuir–Szyszkowski model have provided higher estimates (see Table 1) for the molecular surface area of surfactin, the deviation from the “true” measured molecular surface area obtained using neutron reflectivity is not severe, particularly for the Langmuir–Szyszkowski model. Nonetheless, the molecular surface area estimated using the Frumkin model is the closest to the one obtained using neutron reflectivity measurements. Furthermore, error analysis proved that the deviation from the measured equilibrium surface tension values is the lowest for the case of the Frumkin model (see Table 2). This further supports the superiority of the modified Frumkin model.

**Table 1:** The estimated maximum adsorption density ($\varGamma_{\infty }$) of surfactin, SDBS and C_14_E_8_ and the corresponding area for each surfactant molecule at the liquid–air interface estimated using the Gibbs equation, the Langmuir–Szyszkowski and the Frumkin models

Surfactant parameter^a^	SDBS	Surfactin	C_14_E_8_
$\varGamma_{\infty ,\,G}$ (µmol m^−2^)	3.19	0.97	2.67
$A_{\,G}$ (Å^2^)	52	171	62
$\varGamma_{\infty ,\,L}$ (µmol m^−2^)	3.33	1.05	2.70
$A_{\,L}$ (Å^2^)	50	158	61.5
$\varGamma_{\infty ,\,F}$ (µmol m^−2^)	3.67	1.16	2.84
$A_{\,F}$ (Å^2^)	45	143	58
$\beta$ (−)	−0.80	2.80	−2.10

^a^The subscripts *G*, *L* and *F* indicate that the parameter was estimated using the Gibbs equation, the Langmuir–Szyszkowski or the Frumkin model, respectively

**Table 2:** Error analysis

Error model [47]	Surfactin			SDBS			C_14_E_8_		
	Langmuir	Gibbs	Frumkin	Langmuir	Gibbs	Frumkin	Langmuir	Gibbs	Frumkin
RMSE^a^ (mN/m)	0.162	0.177	0.129	0.413	0.228	0.141	0.712	0.761	0.583
SSE^b^ (mN/m)^2^	0.236	0.281	0.150	0.681	0.157	0.080	3.548	3.474	2.380
CFEF^c^ (mN/m)	0.007	0.008	0.004	0.0174	0.004	0.002	0.098	0.096	0.065
MPSD^d^ (mN/m)	2.16 × 10^−4^	2.36 × 10^−4^	1.33 × 10^−4^	4.75 × 10^−4^	1.34 × 10^−4^	5.01 × 10^−5^	0.003	0.003	0.002
ARE^e^ (−)	0.034	0.039	0.032	0.045	0.023	0.015	0.110	0.109	0.086
EABS^f^ (mN/m)	1.137	1.348	1.100	1.751	0.829	0.600	4.100	4.050	3.200
APE^g^ (−)	0.310	0.355	0.294	0.744	0.450	0.252	1.227	1.365	0.954

^a^Residual root mean square error (RMSE) $= \sqrt {\frac{1}{n - 2}\sum\limits_{i}^{n} {\left( {\gamma_{\exp }^{e} - \gamma_{\text{cal}}^{e} } \right)^{2} } }$

^b^Sum of the squares of the errors (SSE) $= \sum\limits_{i}^{n} {\left( {\gamma_{\exp }^{e} - \gamma_{\text{cal}}^{e} } \right)^{2} }$

^c^Composite fractional error function (CFEF) $= \sum\limits_{i}^{n} {\frac{{\left( {\gamma_{\exp }^{e} - \gamma_{\text{cal}}^{e} } \right)^{2} }}{{\gamma_{\exp }^{e} }}}$

^d^The derivative of the Marquardt’s percent standard deviation (MPSD) $= \sum\limits_{i}^{n} {\left( {\frac{{\gamma_{\exp }^{e} - \gamma_{\text{cal}}^{e} }}{{\gamma_{\exp }^{e} }}} \right)^{2} }$

^e^Average relative error (ARE) $= \sum\limits_{i}^{n} {\left| {\frac{{\gamma_{\exp }^{e} - \gamma_{\text{cal}}^{e} }}{{\gamma_{\exp }^{e} }}} \right|}$

^f^Sum of the absolute errors (EABS) $= \sum\limits_{i}^{n} {\left| {\gamma_{\exp }^{e} - \gamma_{\text{cal}}^{e} } \right|}$

^g^Average percentage errors (APE) $= \frac{{\sum\limits_{i}^{n} {\left| {\left( {\gamma_{\exp }^{e} - \gamma_{\text{cal}}^{e} } \right)/\gamma_{\exp }^{e} } \right|} }}{n} \times 100$

The regression of $\gamma_{2}^{e} - C_{2}$ data using the Frumkin model also provided an estimate for the lateral interaction parameter (β) between the adsorbed surfactin molecules. The estimated β value is 2.8, suggesting a net attractive interaction between the adsorbed molecules. It has been reported that the peptide ring of surfactin adopts a conformation resembling a horse saddle [1] or a ball-like structure [35]. Such a configuration might promote a strong hydrophobic interaction between the hydrophobic portions of surfactin. Screening the negative charges on the aspartic acid (Asp) and the glutamic acid (Glu) of surfactin due to the presence of high concentration (>38.5 mM) of the counter-ion (Na^+^) might also play a role in reducing the electrostatic repulsive forces and bringing surfactin molecules closer to each other and thus further facilitates the hydrophobic interaction.

**Fig. 2:**
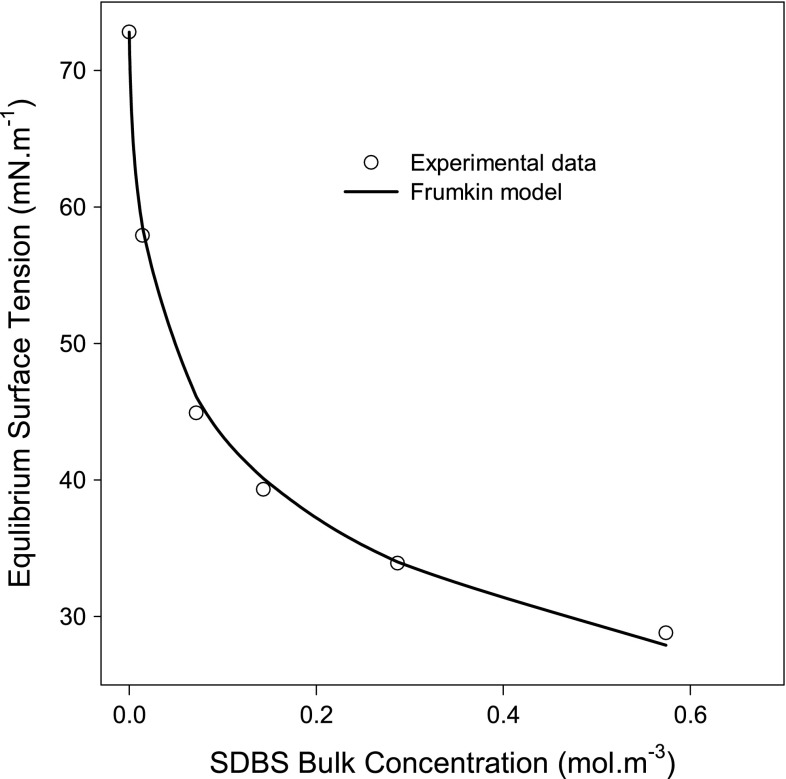
Regression of SDBS $\gamma^{e} - C$ data using the Frumkin model (the coupled Eqs. 3 and 4). The estimated maximum adsorption density ($\varGamma_{\infty }$) and the area occupied by an SDBS molecule at the liquid–air interface are shown in Table 1

To compare the adsorption of this interesting anionic biosurfactant to that of synthetic anionic surfactants, SDBS adsorption at the liquid–air interface has been investigated. The equilibrium surface tension versus SDBS concentration is plotted in Fig. 2. The experimental $\gamma^{e} - C$ data were regressed using the Frumkin model (Eqs. 3 and 4). The estimated $\varGamma_{\infty }$ is 3.67 µmol m^−2^, corresponding to an area per SDBS molecule of 45 Å^2^, which is almost one-third of that occupied by a surfactin molecule. Interestingly, the ratio of the molecular surface area of the two anionic surface active molecules at the liquid–air interface is very similar to their molecular weight ratio. However, unlike the attractive interaction between the adsorbed surfactin molecules, SDBS molecules at the interface experience a repulsive interaction (β = −0.8). Nonetheless, the low value of β indicates that the repulsion between the adsorbed SDBS molecules is relatively weak. This could be caused by the charge screening due to the presence of a high concentration of the counter-ion [39].

The molecular surface area of SDBS estimated using the Frumkin model is strikingly the same as that reported using NR by He *et al*. [40] for one of the SDBS isomers adsorption at the liquid–air interface. However, the molecular surface area of SDBS at the interface estimated using the Gibbs or Langmuir-Szyszkowski models (Eqs. 1 and 2) are slightly higher (52 and 50 Å^2^, respectively). These molecular surface areas are close to that (≈53 Å^2^) reported by Zhang *et al*. [41] for SDBS adsorption at the water–air interface. Nonetheless, unlike the case of Zhang *et al*. [41] in which the adsorption of SDBS took place from water, the adsorption of SDBS under our experimental condition takes place from a solution containing a high concentration (>38.5 mM) of the counter-ion (Na^+^). Since the presence of counter-ions enhances surfactant adsorption [42] (tighter packing), the molecular surface area estimated using the Frumkin model is likely more accurate. This assertion is supported by the error analysis shown in Table 2, where the Frumkin model shows better agreement (lower error) with the experimental data. Although the Frumkin model tracks the experimental data better than the other two models (based on error analysis), the differences between the molecular surface areas estimated using the three different models are not significant (see Table 1).

**Fig. 3:**
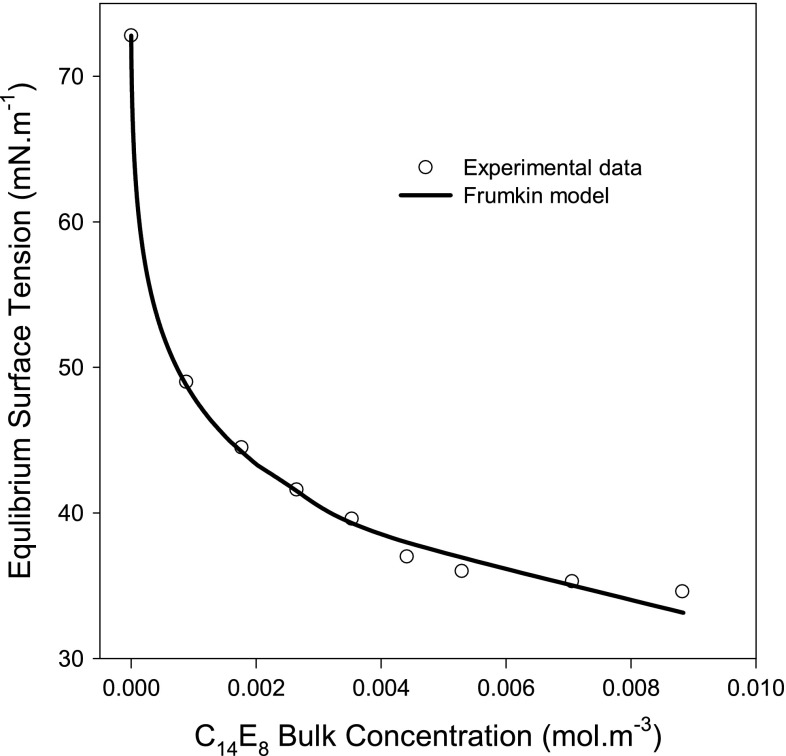
Regression of C_14_E_8_ $\gamma^{e} - C$ data using the Frumkin model (the coupled Eqs. 3 and 4). The estimated maximum adsorption density ($\varGamma_{\infty }$) and the area occupied by C_14_E_8_ molecule at the liquid–air interface are shown in Table 1

In addition to comparing the adsorption of surfactin to that of the synthetic anionic SDBS surfactant, its adsorption is also contrasted to that of the synthetic nonionic surfactant, C_14_E_8_. To enable such benchmarking, the adsorption of C_14_E_8_ at the liquid–air interface was studied. The equilibrium surface tensions at different bulk concentrations of the nonionic surfactant are plotted in Fig. 3. The experimental $\gamma^{e} - C$ data were regressed using the Frumkin model (Eqs. 3 and 4) as shown in Fig. 3. The estimated value of $\varGamma_{\infty }$ is 2.84 µmol m^−2^, corresponding to a molecular surface area of 58 Å^2^. Lu *et al*. [43] studied the adsorption of C_12_E_8_ at the water–air interface using NR from the surfactant micellar solution and reported a molecular surface area of 62 ± 3 Å^2^. C_12_E_8_ is a very close homologue (only two carbon atoms shorter) to C_14_E_8_ and their molecular surface areas are expected to be insignificantly different. This expectation is supported by the findings of Lu *et al*. [43] who reported no significant changes in the areas occupied by C_12_E_n_ at the water–air interface upon increasing the number of the ethoxy group (E) by 1 (e.g., C_12_E_5_ and C_12_E_6_).

The area occupied by a C_14_E_8_ molecule at the liquid–air interface is about 40 % of the area occupied by a surfactin molecule at the interface while the molecular weight ratio of surfactin to C_14_E_8_ is ~1.9:1. Thus, unlike the case of SDBS, the ratio of the area occupied by a C_14_E_8_ molecule to that occupied by a surfactin molecule deviates from the molecular weight ratio of the two surfactants.

The estimated $\varGamma_{\infty }$ value (2.84 µmol m^−2^) for C_14_E_8_ adsorption at the liquid–air interface using the Frumkin model reported in this study is close to that (2.71 µmol m^−2^) reported by Lin *et al*. [33] who also used the same model to estimate $\varGamma_{\infty }$ from $\gamma^{e} - C$ data. However, Karakashev *et al*. [44] have reported a slightly higher $\varGamma_{\infty }$ value (3.33 µmol m^−2^); this value was also estimated from the regression of $\gamma^{e} - C$ data using the Frumkin model. Furthermore, Ueno *et al*. [20] studied the adsorption of C_14_E_8_ at the water–air interface and estimated a similar $\varGamma_{\infty }$ value of 3.33 µmol m^−2^ from the $\gamma^{e} - C$ data using the Gibbs equation. We have also used Gibbs equation and estimated a $\varGamma_{\infty }$ value of 2.67 µmol m^−2^. Additionally, we have used the Langmuir-Szyszkowski model and calculated a value equivalent to 2.70 µmol m^−2^ for $\varGamma_{\infty }$. These findings reveal that the estimated $\varGamma_{\infty }$ values for C_14_E_8_ adsorption at the liquid–air interface reported in this work using the three prediction models are very close (within 6 %). Nonetheless, the Frumkin model has the lowest error, giving it a slight advantage over the Gibbs equation and the Langmuir–Szyszkowski model.

Another important parameter obtained from the regression of $\gamma^{e} - C$ data using the Frumkin model is the lateral interaction parameter (β) between the adsorbed C_14_E_8_ molecules at the interface. Since C_14_E_8_ molecules are neutral (uncharged), the hydrophobic attraction (positive value for β) between the C_14_E_8_ molecules adsorbed at the interface is expected to be significant. However, the estimated value for β is −2.1, suggesting a repulsive interaction between the adsorbed C_14_E_8_ molecules. Negative values for β have been also reported by other researchers for other nonionic surfactants adsorption at liquid–air interfaces [45, 46]. Such non-physical values of β have motivated Fainerman *et al*. [7] to propose that, in such cases, β might be considered as only a fitting parameter with no physical meaning.

## Conclusions

The Frumkin model seems to provide a more accurate estimation of the maximum adsorption density of the biosurfactant, surfactin, as well as of the other two synthetic surfactants. The lateral interaction between the adsorbed surfactin molecules is estimated to be attractive despite the fact that surfactin carries two permanent negative charges at pH 8. Such attraction is probably promoted by the conformation of surfactin, which might have brought the hydrophobic moieties of surfactin closer to each other. Screening the negative charges on the two amino acids (Glu and Aps) of surfactin by the counter-ion (Na^+^) would play a role in minimizing the Debye length and this may further enhance the hydrophobic attraction between the hydrophobic portions of the adsorbed surfactin molecules. Unlike the attractive interaction between the interfacially assembled anionic surfactin molecules, the interaction between the adsorbed anionic SDBS molecules was estimated to be repulsive. Despite the different modes of lateral interaction between the adsorbed surfactin and SDBS molecules, the ratio of the area occupied by a surfactin molecule to that occupied by an SDBS molecule is comparable to their molecular mass ratio. Such correlation, however, was not established between surfactin and the nonionic C_14_E_8_ surfactant.
